# Supplementary material for: Search for ambient superconductivity in the Lu-N-H system
Source: Nat Commun. 2023 Sep 4;14:5367. doi: 10.1038/s41467-023-41005-2 (PMC10477194; doi:10.1038/s41467-023-41005-2)
Supplement: Supplementary file 1 — Supplementary Information [file 41467_2023_41005_MOESM1_ESM.pdf]

Supplementary Information
Search for ambient superconductivity in the Lu–N–H system

Pedro P. Ferreira[a],[1,2] Lewis J. Conway[a],[3,4] Alessio Cucciari,[5,6] Simone Di Cataldo,[7,5] Federico Giannessi,[5,6] Eva Kogler,[2] Luiz T. F. Eleno,[1] Chris J. Pickard,[3,4,†] Christoph Heil,[2,‡] and Lilia Boeri[5,6,§]

[1]*Universidade de São Paulo, Escola de Engenharia de Lorena, DEMAR, 12612-550, Lorena, Brazil*
[2]*Institute of Theoretical and Computational Physics, Graz University of Technology, NAWI Graz, 8010 Graz, Austria*
[3]*Department of Materials Science and Metallurgy, University of Cambridge, Cambridge CB30FS, United Kingdom*
[4]*Advanced Institute for Materials Research, Tohoku University, Sendai 980-8577, Japan*
[5]*Dipartimento di Fisica, Sapienza Università di Roma, 00185 Rome, Italy*
[6]*Enrico Fermi Research Center, Via Panisperna 89 A, 00184, Rome, Italy*
[7]*Institut für Festkörperphysik, Wien University of Technology, 1040 Wien, Austria*
(Dated: August 17, 2023)

I. SUPPLEMENTARY METHODS

A. Ephemeral Data Derived Potentials

Ephemeral Data Derived Potentials (EDDPs) can be used to accelerate crystal structure prediction. We trained potentials using the iterative approach described in reference [1]. In summary, this begins with 1000 single-point-energy calculations of randomly generated structures on which an EDDP is trained. In each iteration, 100 local minima are found by random searching using the current EDDP, and single-point-energies are calculated for 10 'shaken' structures in the vicinity of each minimum. At the end of each iteration, a new EDDP is trained.

For Lu + N + H potentials, we used 5 iterations and local minima found at pressures randomly chosen between 0 and 10 GPa. The form of the potential is naturally cut-off at a distance of 6Å, containing 5 polynomials and a neural network containing a single layer with 5 nodes. The structures in the training data contained between 0-2, 0-4, and 0-12 Lu, N, and H, respectively. By construction, the atoms can come in close contact, such as 0.5Åfor H-H and 2.5ÅLu-Lu, to sample unfavorable high-energy configurations.

B. Convergence tests

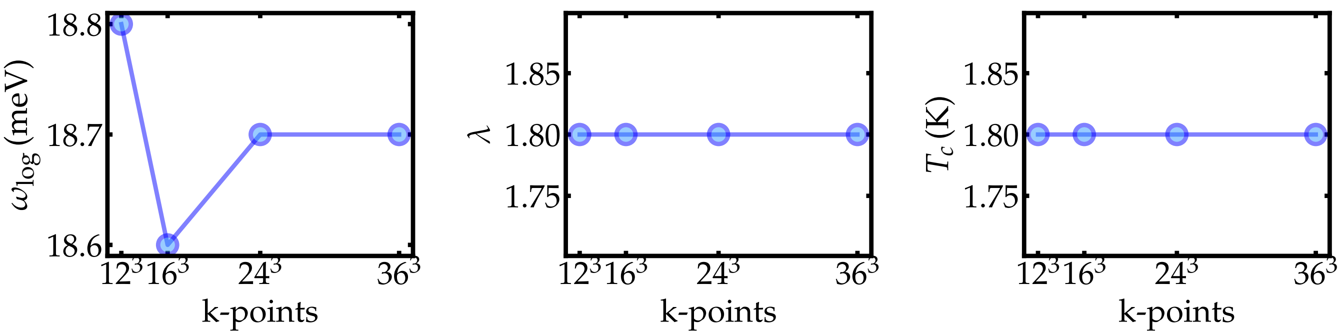

Supplementary Figure 1: Convergence test for B4-$Fm\bar{3}m$-LuH_3 of the electron-phonon properties with respect to the **k**-grid. The $\omega_{\log}$, λ, and T_c were obtained by integrating the Eliashberg function only on real frequencies.

---

[a] These authors contributed equally.
[†] Corresponding author: cjp20@cam.ac.uk
[‡] Corresponding author: christoph.heil@tugraz.at
[§] Corresponding author: lilia.boeri@uniroma1.it

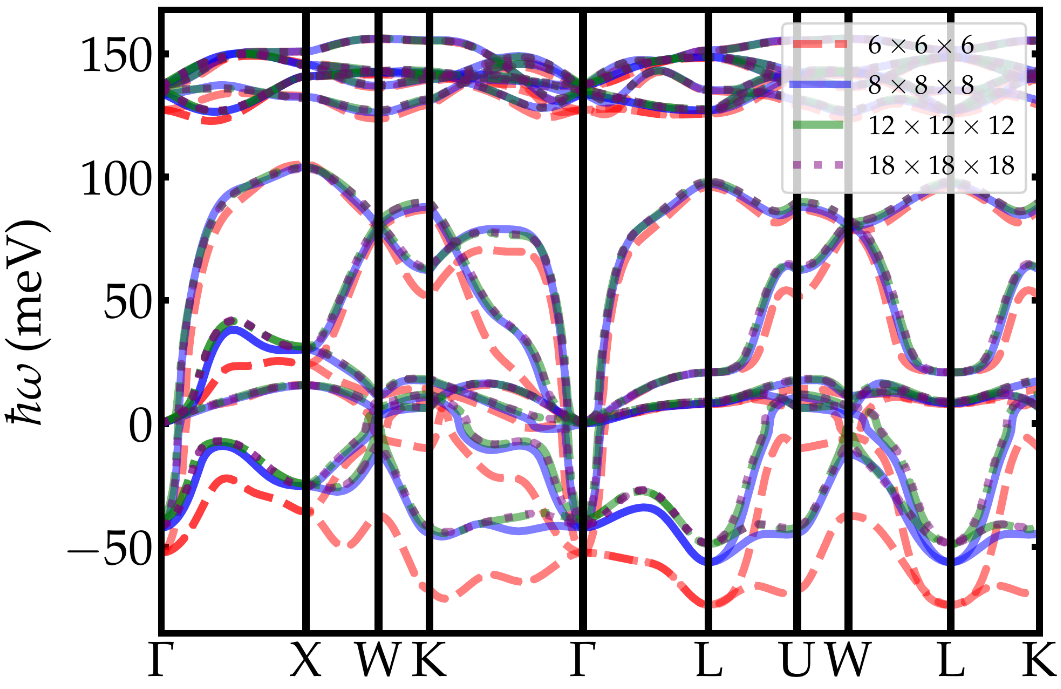

Supplementary Figure 2: Convergence test for B4-$Fm\overline{3}m$-LuH_3 of the phonon dispersion with respect to the **k**-grid employed to generate the self-consistent charge density.

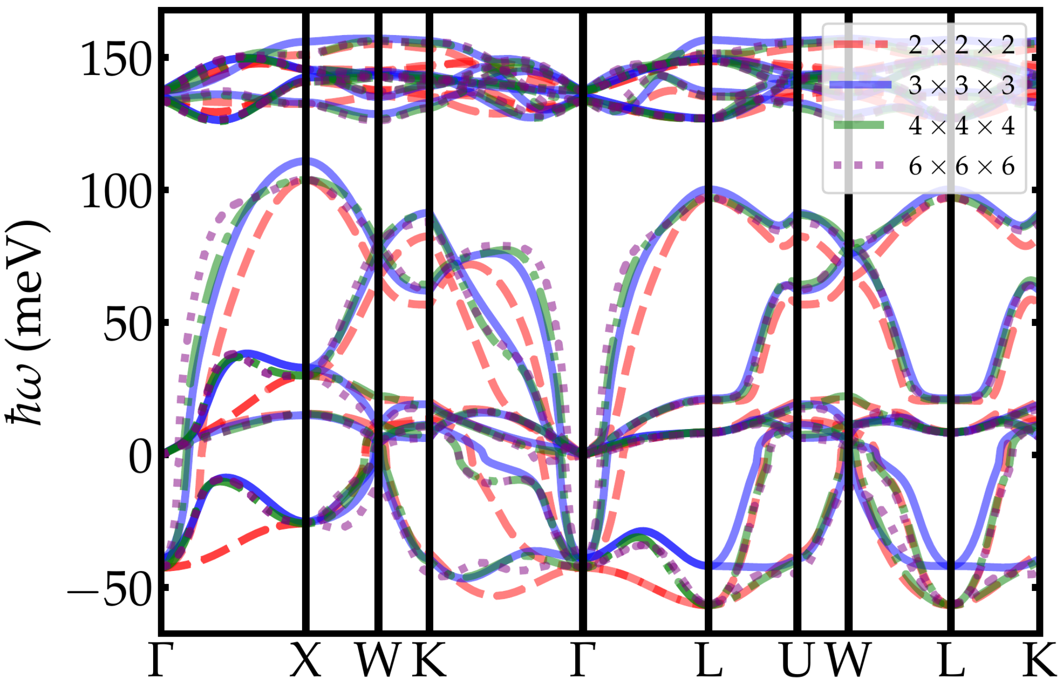

Supplementary Figure 3: Convergence test for B4-$Fm\overline{3}m$-LuH_3 of the phonon dispersion with respect to the **q**-grid.

C. Convex hull

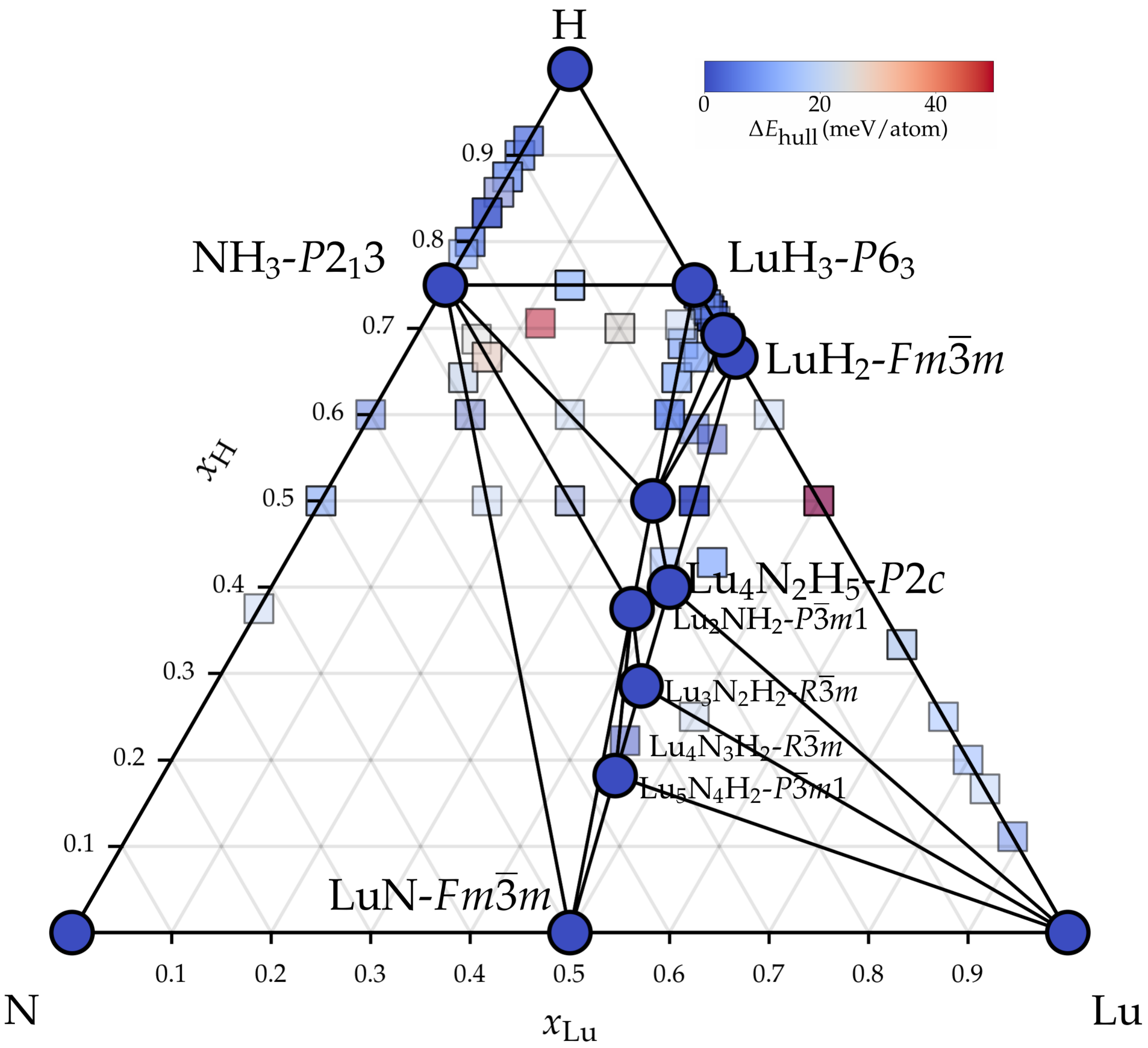

Supplementary Figure 4: Convex hull for the Lu–N–H ternary system at 10 GPa.

II. SUPPLEMENTARY NOTES

A. Crystallographic data

Supplementary Table 1: Crystallographic data for selected binary phases with LuH, LuH_2 and LuH_3 composition predicted at ambient pressure.

ID	Comp.	SG	ΔE_{hull} (meV/atom)	Lattice parameters (Å)	Sites	x	y	z
B1	LuH	$F\bar{4}3m$	102	$a = 5.019$	Lu(4*d*)	0.2500	0.2500	0.7500
					H(4*a*)	0.0000	0.0000	0.0000
B2	LuH	$Fm\bar{3}m$	222	$a = 4.794$	Lu(4*a*)	0.0000	0.0000	0.0000
					H(4*b*)	0.0000	0.0000	0.5000
B3	LuH_2	$Fm\bar{3}m$	0	$a = 4.999$	Lu(4*a*)	0.0000	0.0000	0.0000
					H(8*c*)	0.2500	0.2500	0.2500
B4	LuH_3	$Fm\bar{3}m$	101	$a = 5.005$	Lu(4*a*)	0.0000	0.0000	0.0000
					H(4*b*)	0.0000	0.0000	0.5000
					H(8*c*)	0.2500	0.2500	0.2500
B5	LuH_3	$P6_3/mmc$	10	$a = 3.528$	Lu(2*c*)	0.3333	0.6667	0.2500
				$c = 6.341$	H(2*a*)	0.0000	0.0000	0.0000
					H(4*f*)	0.3333	0.6667	0.5955

Supplementary Table 2: Crystallographic data for ternary metallic phases within 50 meV/atom from the convex hull predicted at 0 GPa

ID	Comp.	SG	ΔE_{hull} (meV/atom)	Lattice parameters	Sites	x	y	z
T1	LuNH	$P\bar{4}3m$	485	$a = 4.990$	Lu(4*e*)	0.2354	0.2354	0.7646
					N(4*e*)	0.2276	0.2276	0.2276
					H(4*e*)	0.3586	0.3586	0.3586
T2	Lu_2NH_2	$P\bar{3}m1$	5	$a = 3.432$	Lu(2*d*)	0.3333	0.6667	0.7658
				$c = 5.647$	N(1*a*)	0.0000	0.0000	0.0000
					H(2*d*)	0.3333	0.6667	0.3852
T3	Lu_2NH_3	$P\bar{3}m1$	20	$a = 3.446$	Lu(2*d*)	0.3333	0.6667	0.7438
				$c = 5.555$	N(1*b*)	0.0000	0.0000	0.5000
					H(1*a*)	0.0000	0.0000	0.0000
					H(2*d*)	0.3333	0.6667	0.1360
T4	Lu_3NH_4	$R\bar{3}m$	6	$a = 3.463$	Lu(3*a*)	0.0000	0.0000	0.0000
				$c = 25.647$	Lu(6*c*)	0.0000	0.0000	0.2183
					N(3*b*)	0.0000	0.0000	0.5000
					H(6*c*)	0.0000	0.0000	0.0834
					H(6*c*)	0.0000	0.0000	0.3035
T5	$Lu_3N_2H_2$	$R\bar{3}m$	2	$a = 3.409$	Lu(3)	0.0000	0.0000	0.5000
				$c = 25.189$	Lu(6*c*)	0.0000	0.0000	0.2731
					N(6*c*)	0.0000	0.0000	0.1118
					H(6*c*)	0.0000	0.0000	0.3587
T6	$Lu_3N_2H_3$	$R\bar{3}m$	14	$a = 3.419$	Lu(3*a*)	0.0000	0.0000	0.0000
				$c = 24.896$	Lu(6*c*)	0.0000	0.0000	0.2240
					N(6*c*)	0.0000	0.0000	0.3879
					H(3*b*)	0.0000	0.0000	0.5000
					H(6*c*)	0.0000	0.0000	0.1366
T7	Lu_4NH_6	$R\bar{3}m$	6	$a = 3.486$	Lu(6)	0.0000	0.0000	0.2090
				$c = 34.306$	Lu(6*c*)	0.0000	0.0000	0.3718
					N(3*a*)	0.0000	0.0000	0.0000
					H(6*c*)	0.0000	0.0000	0.1456
					H(6*c*)	0.0000	0.0000	0.2714
					H(6*c*)	0.0000	0.0000	0.4352
T8	$Lu_4N_2H_5$	$C2/m$	2	$a = 12.694$	Lu(4*i*)	0.1305	0.0000	0.9669
				$c = 5.945$	Lu(4*i*)	0.1313	0.5000	0.4607
				$\beta = 117.813$	N(4*i*)	0.2491	0.0000	0.7537
					H(2*b*)	0.0000	0.5000	0.0000
					H(4*i*)	0.0599	0.5000	0.7157
					H(4*i*)	0.0621	0.0000	0.2442
T9	$Lu_4N_2H_5$	$P2/c$	0	$a = 5.609$	Lu(4*g*)	0.2600	0.1221	0.0807
				$b = 6.866$	Lu(4*g*)	0.2640	0.3721	0.5857
				$c = 5.951$	N(2*f*)	0.5000	0.1243	0.7500
				$\alpha = \gamma = 90.000$	N(2*f*)	0.5000	0.3739	0.2500
				$\beta = 90.062$	H(2*e*)	0.0000	0.1252	0.7500
					H(4*g*)	0.1195	0.3835	0.9218
					H(4*g*)	0.1240	0.1364	0.4077
T10	$Lu_4N_3H_2$	$R\bar{3}m$	2	$a = 3.398$	Lu(6*c*)	0.0000	0.0000	0.2070
				$c = 33.431$	Lu(6*c*)	0.0000	0.0000	0.3790
					N(3*b*)	0.0000	0.0000	0.5000
					N(6*c*)	0.0000	0.0000	0.0840
					H(6*c*)	0.0000	0.0000	0.3145
T11	Lu_5NH_8	$P\bar{3}m1$	6	$a = 3.497$	Lu(1*b*)	0.0000	0.0000	0.5000
				$c = 14.337$	Lu(2*d*)	0.3333	0.6667	0.9081
					Lu(2*d*)	0.3333	0.6667	0.2972
					N(1*a*)	0.0000	0.0000	0.0000
					H(2*c*)	0.0000	0.0000	0.3481
					H(2*d*)	0.3333	0.6667	0.1477
					H(2*d*)	0.3333	0.6667	0.4481
					H(2*d*)	0.3333	0.6667	0.7565
T12	$Lu_5N_4H_2$	$P\bar{3}m1$	2	$a = 3.389$	Lu(1*b*)	0.0000	0.0000	0.5000
				$c = 13.896$	Lu(2*d*)	0.3333	0.6667	0.6952
					Lu(2*d*)	0.3333	0.6667	0.1098
					N(2*c*)	0.0000	0.0000	0.2024
					N(2*d*)	0.3333	0.6667	0.4008
					H(2*d*)	0.3333	0.6667	0.9551

B. Simulated XRD diffractograms

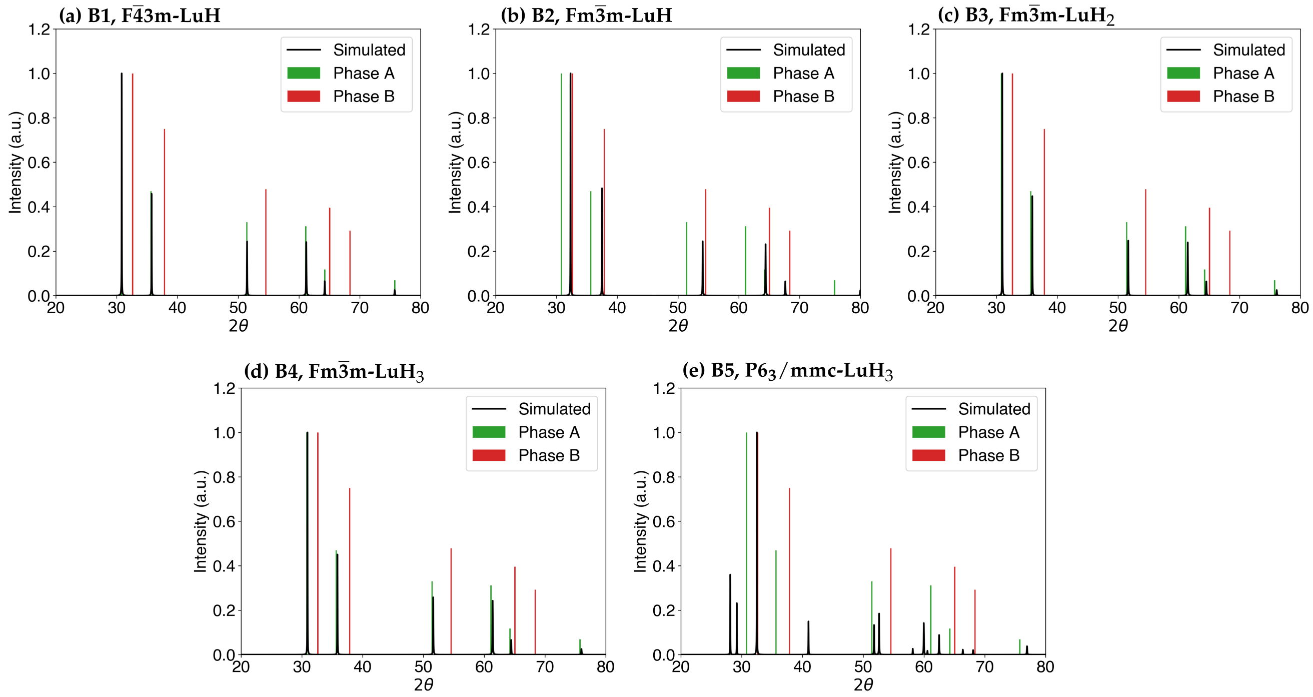

Supplementary Figure 5: Simulated XRD diffractogram (black lines) compared to the obtained Bragg peaks of Phase A (green lines) and Phase B (red lines) in Dasenbrock-Gammon *et al.* [2] for the binaries (a) B1, $F\bar{4}3m$-LuH; (b) B2, $Fm\bar{3}m$-LuH; (c) B3, $Fm\bar{3}m$-LuH$_2$; (d) B4, $Fm\bar{3}m$-LuH$_3$; and (e) B5, $P6_3/mmc$-LuH$_3$.

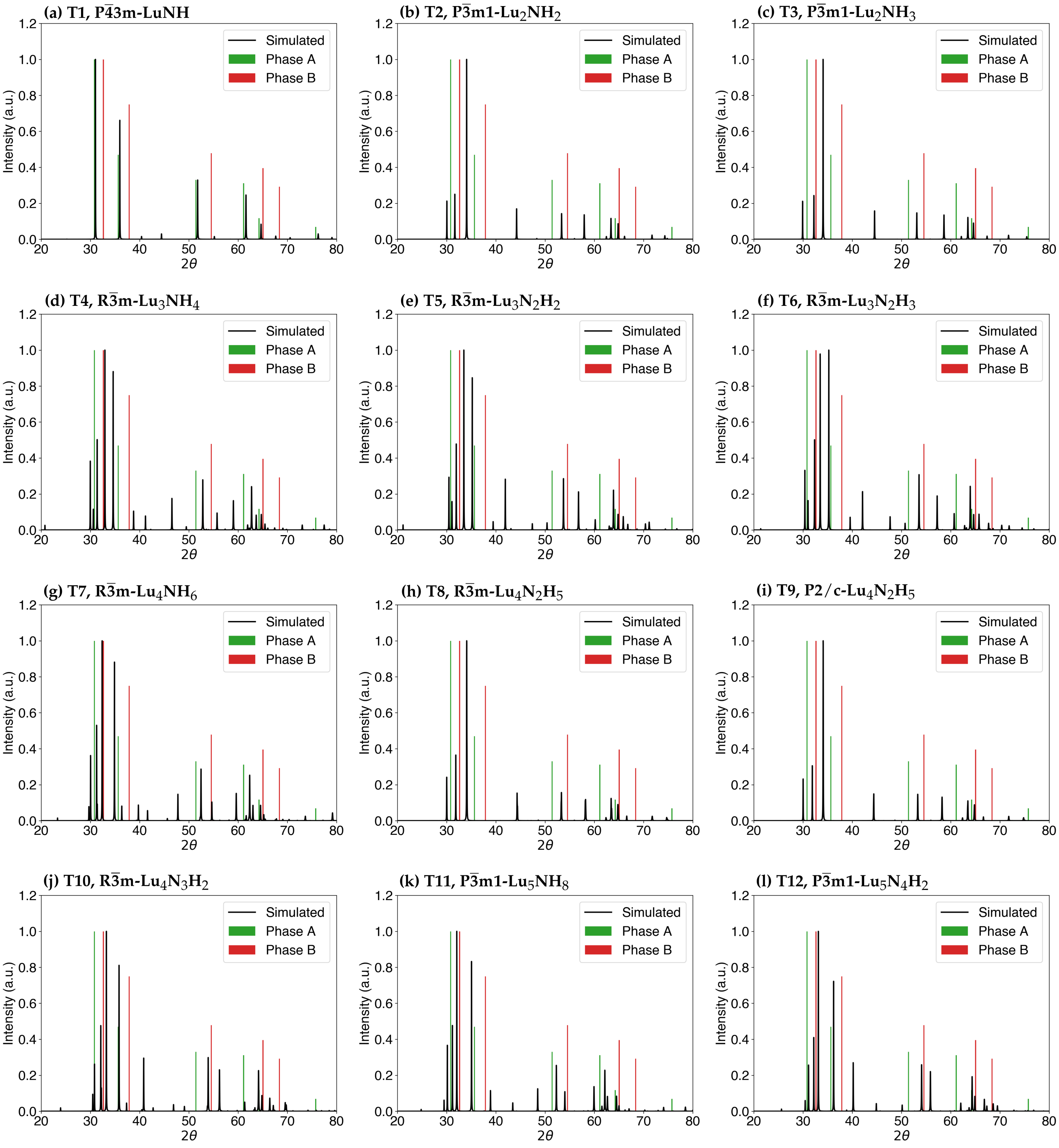

Supplementary Figure 6: Simulated XRD diffractogram (black lines) compared to the obtained Bragg peaks of Phase A (green lines) and Phase B (red lines) in Dasenbrock-Gammon *et al.* [2] for the ternaries (a) (a) T1, $P\bar{4}3m$-LuNH; (b) T2, $P\bar{3}m1$-Lu_2NH_2; (c) T3, $P\bar{3}m1$-Lu_2NH_3; (d) T4, $R\bar{3}m$-Lu_3NH_4; (e) T5, $R\bar{3}m$-$Lu_3N_2H_2$; (f) T6, $R\bar{3}m$-$Lu_3N_2H_3$; (g) T7, $R\bar{3}m$-Lu_4NH_6; (h) T8, $R\bar{3}m$-$Lu_4N_2H_5$; (i) T9, $P2/c$-$Lu_4N_2H_5$; (j) T10, $R\bar{3}m$-$Lu_4N_3H_2$; (k) T11, $P\bar{3}m1$-Lu_5NH_8; and (l) T12, $P\bar{3}m1$-$Lu_5N_4H_2$.

C. Raman data

Supplementary Table 3: Comparison of the reported Raman frequencies of compound A and B in Ref. [2] and the calculated Raman active modes at Γ for B1-LuH$F\bar{4}3m$, B2-LuH$Fm\bar{3}m$, B3-LuH_2-$Fm\bar{3}m$, B4-LuH_3-$Fm\bar{3}m$, and T1-LuNH-P$\bar{4}$3m. All frequencies are given in units of cm^{-1}.

Compound A Ref. [2]	97	112	121	128	138	148	193	214	237	252	1202	1239
Compound B Ref. [2]	115	187	354	426	532							
B1-LuH$F\bar{4}3m$	none											
B2-LuH$Fm\bar{3}m$	none											
B3-LuH_2-$Fm\bar{3}m$	1176											
B4-LuH_3-$Fm\bar{3}m$	1078											
T1-LuNH-P$\bar{4}$3m	49	113	114	143	339	349	349	380	1059	1075	1366	1921

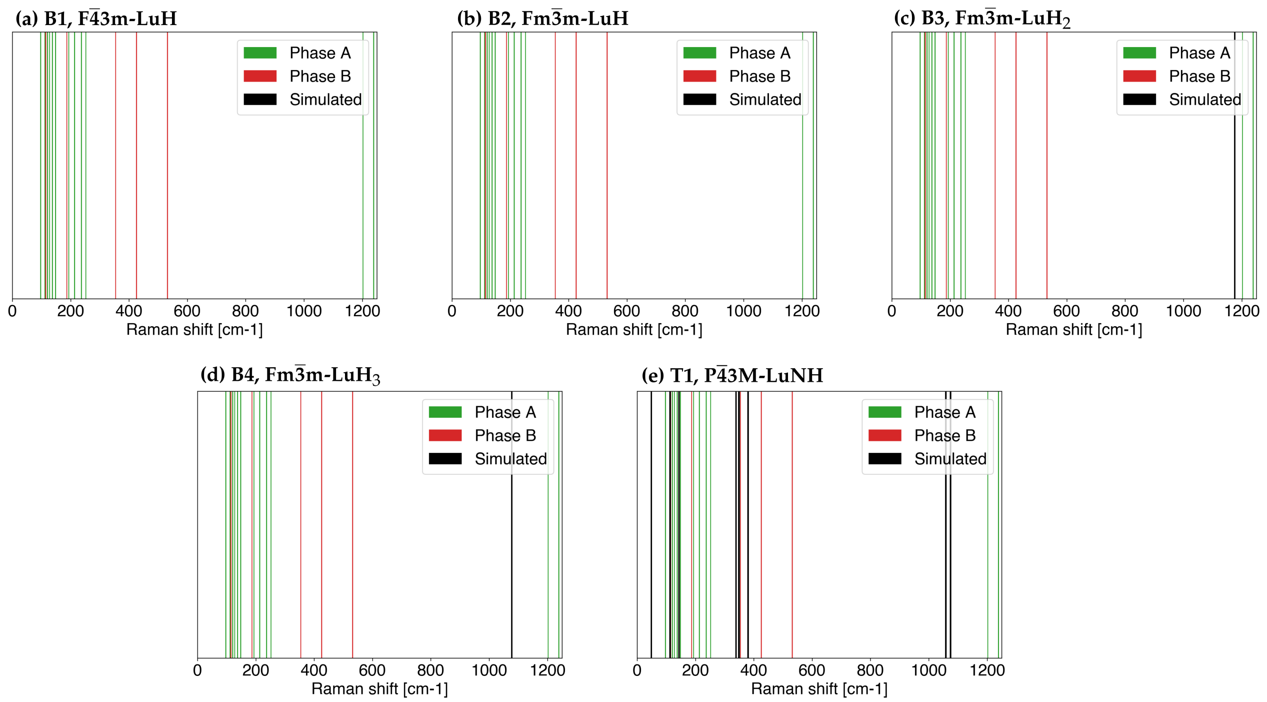

Supplementary Figure 7: Calculated Raman active modes at Γ (black lines) compared to the obtained Raman frequencies of Phase A (green lines) and Phase B (red lines) in Dasenbrock-Gammon *et al.* [2] for (a) B1, $F\bar{4}3m$-LuH; (b) B2, $Fm\bar{3}m$-LuH; (c) B3, $Fm\bar{3}m$-LuH_2; (d) B4, $Fm\bar{3}m$-LuH_3; and (e) T1, $P\bar{4}3m$-LuNH.

D. Electronic structure

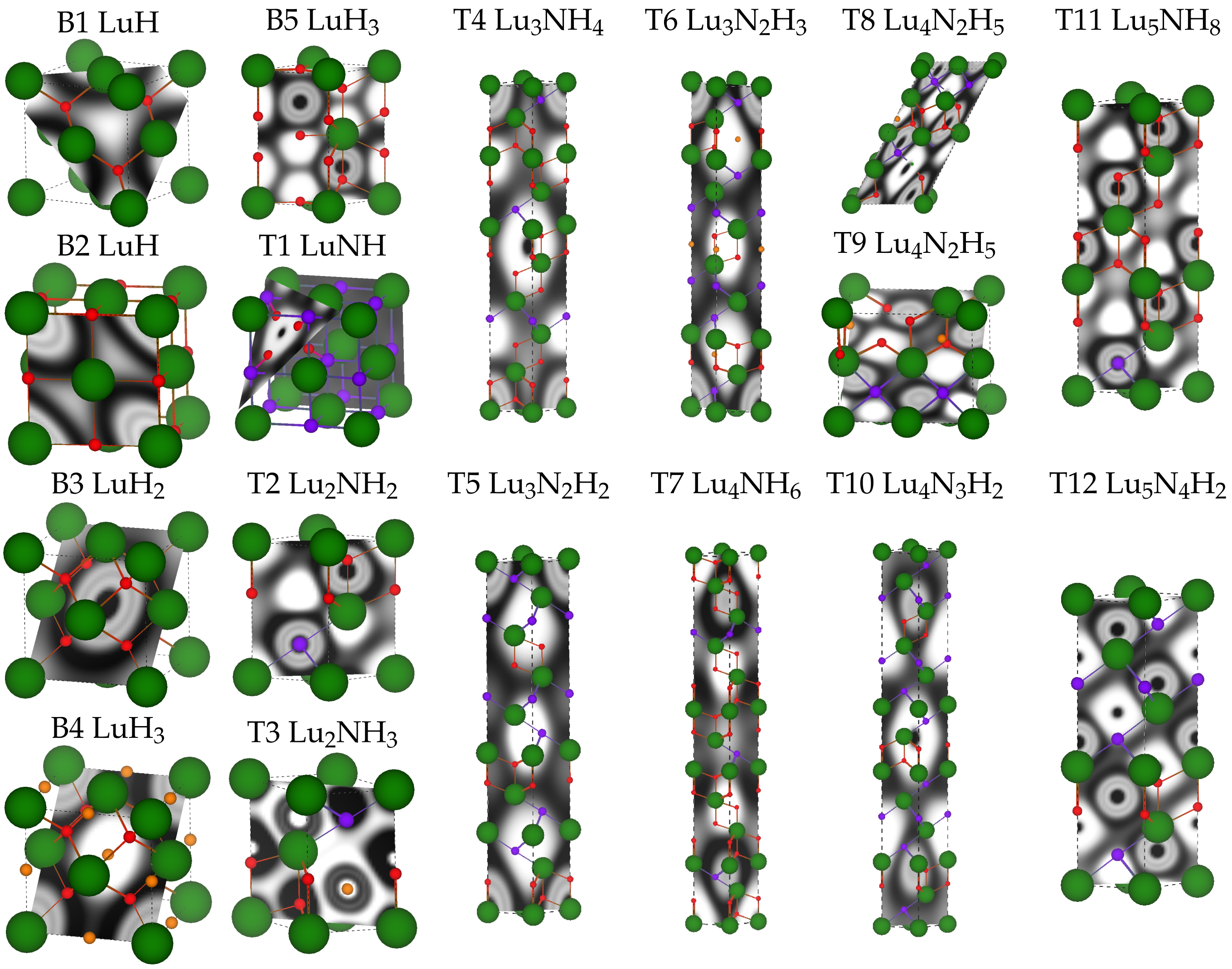

Supplementary Figure 8: Electron localization functions (ELF) of the best candidates for SC in Lu–N–H ternary system as listed in Tab. 1. Lu, N, H, and H in octahedral sites are indicated as large green, medium purple, small red, and small orange spheres, respectively.

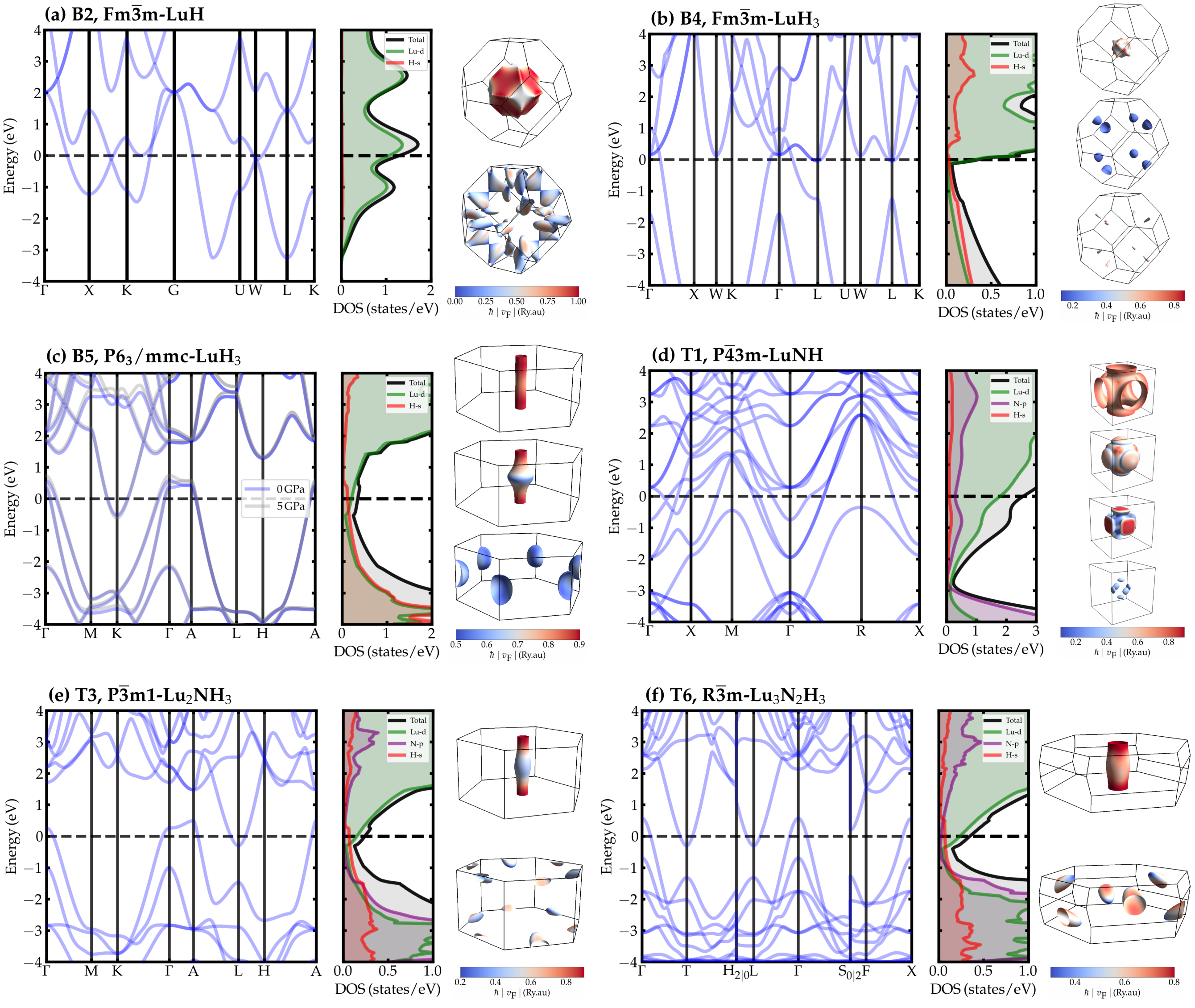

Supplementary Figure 9: Electronic band structure, density of states (DOS), and Fermi surface for (a) B2, $Fm\bar{3}m$-LuH, (b) B4, $Fm\bar{3}m$-LuH_3, (c) B5, $P6_3/mmc$-LuH_3, (d) T1, $P\bar{4}3m$-LuNH, (e) T3, $P\bar{3}m1$-Lu_2NH_3, and (f) T6, $R\bar{3}m$-$Lu_3N_2H_3$.

E. Superconductivity

In this section, we report the details of the effective model for superconductivity discussed in Sec. V and Fig. 4 of the main manuscript. Specifically, we estimate the critical temperatures through the semi-empirical McMillan-Allen-Dynes formula [3].

$$k_B T_c^{AD} = \frac{\omega_{log}}{1.2} \exp\left[- \frac{1.04\,(1+\lambda)}{\lambda(1-0.62\,\mu^*) - \mu^*} \right], \tag{1}$$

where ω_{log} is the logarithmic averaged phonon frequency, λ is the electron-phonon coupling strength, and μ^* is the Morel-Anderson pseudopotential [4].

Moreover, λ was expressed according to the McMillan-Hopfield's formula [5],

$$\lambda = \frac{N(E_F)\, I^2}{M\,\omega^2} \equiv \frac{\eta}{\omega^2}, \tag{2}$$

assuming $\omega = \omega_{log}$. The collected data from the literature for λ and ω_{log} for selected hydrides are reported in Supplementary Table 4, while in Tab. 1 of the main manuscript is reported the data obtained for the newly-predicted Lu–N–H structures.

Supplementary Figures. 10 and 11 show the plots for λ and η.

Supplementary Table 4: Superconducting properties of selected hydrides from literature. To make the comparison with our data consistent, the superconducting critical temperatures (T_c^{AD}) reported in this table were re-computed by employing the semi-empirical McMillan-Allen-Dynes formula with $\mu^* = 0.1$ using the ω_{log} and λ collected in the literature, as cited in the "Ref" column.

Comp.	SG	P (GPa)	ω_{log} (meV)	λ	η (10^4 meV^2)	T_c^{AD} (K)	T_c^{Ref} (K)	Ref.
CrH	P6$_3$/m	0	29	0.67	0.06	11	11	[6]
$PdCuH_2$	Pm$\bar{3}$m	0	28	1.90	0.15	45	45	[7]
$LaBeH_8$	Fm$\bar{3}$m	50	77	2.19	1.31	137	167	[8]
$BaSiH_8$	Fm$\bar{3}$m	50	72	1.02	0.88	60	84	[9]
$LaBH_8$	Fm$\bar{3}$m	75	91	1.06	0.53	80	96	[10]
$SrSiH_8$	Fm$\bar{3}$m	100	65	2.02	0.86	109	169	[9]
LuH_6	Im$\bar{3}$m	100	65	3.60	1.51	145	273	[11]
LuH_6	Im$\bar{3}$m	200	118	1.77	2.46	180	250	[11]
SH_3	Im$\bar{3}$m	200	115	2.19	2.90	204	204	[12]
TbH_{10}	Fm$\bar{3}$m	250	106	2.50	2.79	202	278	[13]
LaH_{10}	Fm$\bar{3}$m	300	104	2.20	2.38	185	234	[14]
YH_6	Im$\bar{3}$m	300	120	1.73	2.49	180	290	[15]
YH_{10}	Fm$\bar{3}$m	300	119	2.41	3.41	223	310	[15]
H	I4$_1$/amd	500	178	1.81	5.75	277	330	[16]
H	R$\bar{3}$m	2000	235	3.06	16.90	495	715	[16]

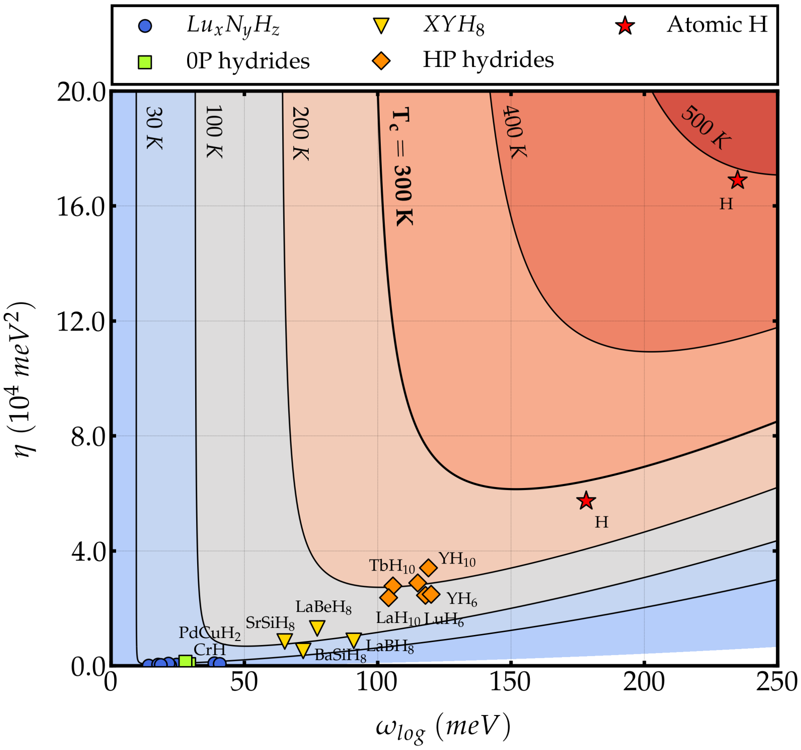

Supplementary Figure 10: η vs $\omega_{\log}$ for different classes of superconducting hydrides. The best Lu–N–H hydrides considered in this work are indicated by blue circles and a selection of other hydrides is included as reference (Supplementary Table 4). Isocontour lines for T_c are plotted according to Eq. 1, with $\mu^* = 0.1$.

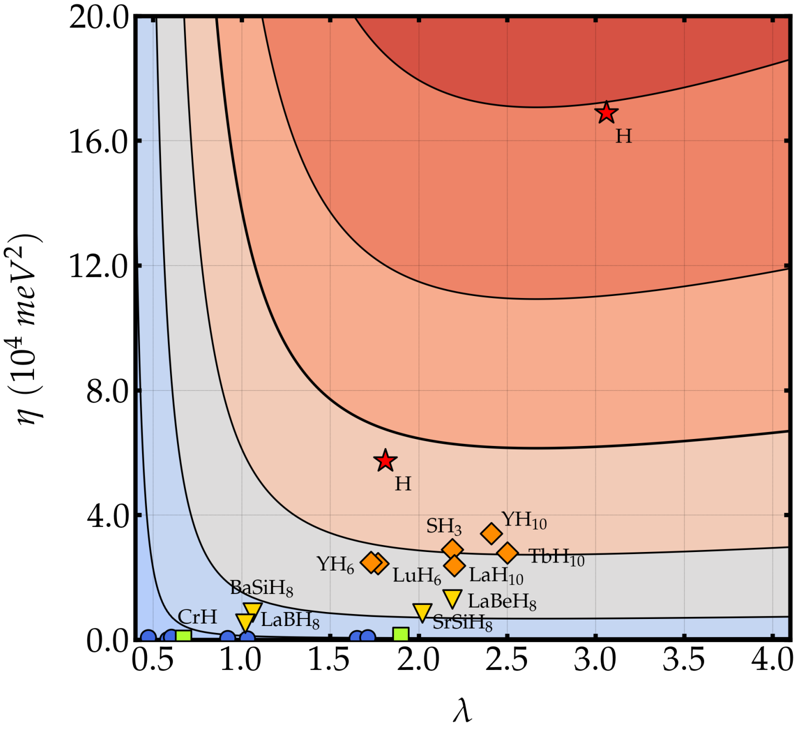

Supplementary Figure 11: η vs λ for different classes of superconducting hydrides. Isocontours refer to T_c calculated using the McMillan-Allen-Dynes equation (Eq. 1), with $\mu^* = 0.1$.

III. SUPPLEMENTARY REFERENCES

[1] Pickard, C. J. Ephemeral data derived potentials for random structure search. *Physical Review B* **106**, 014102 (2022).

[2] Dasenbrock-Gammon, N. *et al.* Evidence of near-ambient superconductivity in a n-doped lutetium hydride. *Nature* **615**, 244–250 (2023). `https://doi.org/10.1038/s41586-023-05742-0`.

[3] Allen, P. B. & Dynes, R. C. Transition temperature of strong-coupled superconductors reanalyzed. *Phys. Rev. B* **12**, 905–922 (1975). `https://link.aps.org/doi/10.1103/PhysRevB.12.905`.

[4] Morel, P. & Anderson, P. W. Calculation of the superconducting state parameters with retarded electron-phonon interaction. *Phys. Rev.* **125**, 1263–1271 (1962).

[5] McMillan, W. L. Transition temperature of strong-coupled superconductors. *Phys. Rev.* **167**, 331–344 (1968). `https://link.aps.org/doi/10.1103/PhysRev.167.331`.

[6] Yu, S. *et al.* Pressure-driven formation and stabilization of superconductive chromium hydrides. *Scientific Reports* **5** (2015). `https://doi.org/10.1038/srep17764`.

[7] Vocaturo, R., Tresca, C., Ghiringhelli, G. & Profeta, G. Prediction of ambient-pressure superconductivity in ternary hydride pdcuhx. *Journal of Applied Physics* **131**, 033903 (2022). `https://doi.org/10.1063/5.0076728`. https://doi.org/10.1063/5.0076728.

[8] Zhang, Z. *et al.* Design principles for high-temperature superconductors with a hydrogen-based alloy backbone at moderate pressure. *Phys. Rev. Lett.* **128**, 047001 (2022). `https://link.aps.org/doi/10.1103/PhysRevLett.128.047001`.

[9] Lucrezi, R., Di Cataldo, S., von der Linden, W., Boeri, L. & Heil, C. In-silico synthesis of lowest-pressure high-t_c ternary superhydrides. *npj Computational Materials* **8** (2022). `https://doi.org/10.1038/s41524-022-00801-y`.

[10] Di Cataldo, S., Heil, C., von der Linden, W. & Boeri, L. Labh$_8$: Towards high-T_c low-pressure superconductivity in ternary superhydrides. *Phys. Rev. B* **104**, L020511 (2021). `https://link.aps.org/doi/10.1103/PhysRevB.104.L020511`.

[11] Song, H. *et al.* High tc superconductivity in heavy rare earth hydrides. *Chinese Physics Letters* **38**, 107401 (2021). `https://iopscience.iop.org/article/10.1088/0256-307X/38/10/107401/meta`.

[12] Duan, D. *et al.* Pressure-induced metallization of dense $(H_2S)_2H_2$ with high-T_c superconductivity. *Scientific Reports* **4**, 6968 (2014). `https://doi.org/10.1038/srep06968`.

[13] Liang, X. *et al.* Potential high-T_c superconductivity in cayh$_{12}$ under pressure. *Phys. Rev. B* **99**, 100505 (2019). `https://link.aps.org/doi/10.1103/PhysRevB.99.100505`.

[14] Flores-Livas, J. A. *et al.* A perspective on conventional high-temperature superconductors at high pressure: Methods and materials. *Physics Reports* **856**, 1–78 (2020). `https://doi.org/10.1016/j.physrep.2020.02.003`.

[15] Heil, C., Cataldo, S. D., Bachelet, G. B. & Boeri, L. Superconductivity in sodalite-like yttrium hydrides. *Phys. Rev. B* 220502 (2019). `https://doi.org/10.1103/PhysRevB.99.220502`.

[16] McMahon, J. M. & Ceperley, D. M. High-temperature superconductivity in atomic metallic hydrogen. *Phys. Rev. B* **84**, 144515 (2011). `https://link.aps.org/doi/10.1103/PhysRevB.84.144515`.
